# A Medical Rarity: Isolated *Toxoplasma gondii* Abscess in the Pituitary Gland of an Immunocompetent Patient

**DOI:** 10.1155/crdi/9943637

**Published:** 2025-07-21

**Authors:** Erlend Moen Taule, Hrvoje Miletic, Rupavathana Mahesparan

**Affiliations:** ^1^Department of Biomedicine, University of Bergen, Bergen, Norway; ^2^Department of Pathology, Haukeland University Hospital, Bergen, Norway; ^3^Department of Clinical Medicine, University of Bergen Faculty of Medicine, Bergen, Norway; ^4^Department of Neurosurgery, Haukeland University Hospital, Bergen, Norway

**Keywords:** neurosurgery, parasite infection, pituitary abscess, toxoplasmosis

## Abstract

The occurrence of sellar toxoplasmosis in the pituitary gland is exceedingly rare, and only a few reports have been published in the literature, primarily impacting immunocompromised patients. We report an intriguing case of a 54-year-old man with an initial asymptomatic panhypopituitarism diagnosed under investigation for urolithiasis. Cranial CT and MRI revealed a large sellar lesion first suspectable of a nonfunctioning pituitary macroadenoma. An endonasal transsphenoidal surgery was performed, and the histopathological diagnosis was surprisingly a pituitary *Toxoplasma gondii* abscess. This case highlights that these types of infections can also occur in immunocompetent patients.

## 1. Introduction

Even though central nervous system infection by *Toxoplasma gondii* is the most common cause of brain mass lesion in human immunodeficiency virus (HIV)–infected patients [1], abscess formation of the pituitary gland has seldom been reported in the literature [2].

*T. gondii* is a zoonotic, intracellular parasite that can exist as tachyzoites, bradyzoites, and oocysts [1]. The finding of *T. gondii* bradycyst in the pituitary is very unusual. Infection of *T. gondii* affecting the pituitary gland was first reported in 1986 by Milligan et al. in a patient with AIDS and congenital toxoplasmosis [3]. Clinically, manifestations are nonspecific and dependent on the growth pattern and pressure to the adjacent structures, but the most common presentations of pituitary abscess have been reported to be headache, visual disturbance, and endocrine abnormalities [4]. Histologically, *T. gondii* abscess in the pituitary gland has been reported as necrotic, scant chronic inflammation, and evidence of cyst and/or tachyzoites [5].

While toxoplasmosis abscesses have been reported in immunosuppressed patients [2–5], instances in immunocompetent individuals remain exceedingly rare. To our knowledge, there has been no documented report of pituitary gland abscess in an immunocompetent patient. Here, we present a case of an immunocompetent patient who developed a *T. gondii* pituitary gland abscess.

## 2. Case Presentation

**Figure 1:**
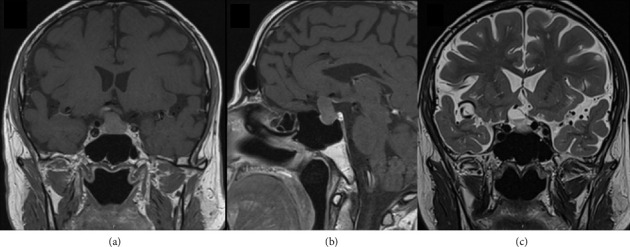
Preoperative MRI brain scan 10/2022. (a) Coronal T1-weighted image showing a hypointense pituitary mass compromising the left sinus cavernous. (b) Sagittal T1 showing the same pituitary mass growing through the diaphragm sellae. (c) Coronal T2-weighted image shows a hyperintense cystic pituitary mass.

A 54-year-old man, previously diagnosed with hypertension, sleep apnea, and non-ST-elevation myocardial infarction. He was diagnosed with cortisol and TSH deficiency during hospitalization for urolithiasis treatment. An MRI of the head in October 2022 showed a 16 × 11 × 21 mm lesion with a cystic component of 11 mm in the sella turcica (Figure 1). The neuroradiological working diagnosis was pituitary macroadenoma with cystic degeneration. The lesion grew through the diaphragm sellae and compromised the left sinus cavernous, with a Knosp Grade 2. It was also compromising the chiasma upward. No other abnormality of the brain was found. The patient was evaluated by an ophthalmologist without signs of visual affection. A follow-up MRI was performed 4 months later and revealed significant growth, and the size was now 19 × 12 × 25 mm. Ophthalmological examination revealed a right temporal visual field deficit.

After discussion in the pituitary multidisciplinary team meeting, the patient was offered surgery. The patient underwent transnasal, transsphenoidal surgery. Peroperatively, a white, odorless purulent material with some necrotic material was seen upon opening the dura and the subsequent exploration. The material was sent for analysis both for microbiology and histopathology. Postoperatively, the patient was continuing hydrocortisone, and he developed transient diabetes insipidus that which was treated with desmopressin. He was discharged home in good condition after 3 days.

**Figure 2:**
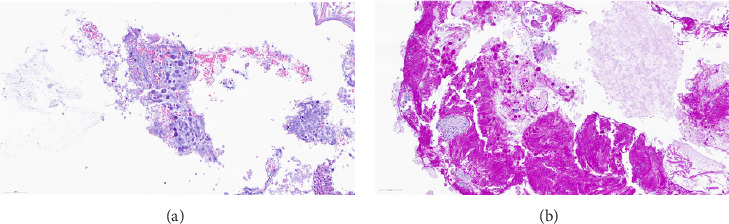
Histology of the resected specimen. (a) Necrotic tissue with *Toxoplasma* cysts, H&E staining. (b) Toxoplasma cysts are positive for PAS. PAS staining. Scale bars 60 μm.

**Figure 3:**
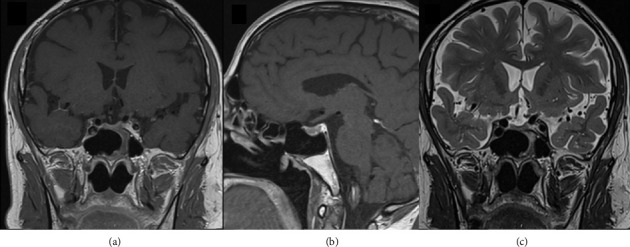
Postoperative MRI brain scan 9/2023. (a) Coronal T1-weighted image shows less compression of the left sinus cavernous. (b) Sagittal T1-weighted image shows relief of pressure on the optic chiasm. (c) Coronal T2-weighted image shows cessation of the preoperative cystic compartment.

Histology on hematoxylin–eosin (H&E) stained sections showed necrotic material with focal infiltration of neutrophilic granulocytes and small oval structures with granular contents compatible with toxoplasmosis cysts (Figure 2). These were confirmed by PAS staining (Figure 2). There were satisfying postoperative MRI brain scans (Figure 3) with less compression of the left sinus cavernous and optic chiasm.

Serologically, there was no evidence of either active or previous inflammation (negative for *T. gondii*-specific antibodies IgG and IgM). The patient had no signs of immunodeficiency in his medical history. Consequently, it was concluded that the patient did not require further treatment for *T. gondii*. A follow-up MRI was conducted 3 months postoperation, with plans for another MRI 1 year after the operation, without any recurrence of the abscess.

He is currently under the care of endocrinologists for hypopituitarism, receiving treatment with somatropin, cortisone, levothyroxine, and testosterone. The patient owned a cat, which may have served as the source of infection.

In addition, we have conducted a comprehensive literature review to gather case reports and case series in order to further investigate this rare condition.

## 3. Discussion

It's estimated that around a third of the world population is infected by *Toxoplasma gondii* [6], with primary infection in immunocompetent patients most often remaining asymptomatic [7]. Central nervous system infection of *T. gondii* commonly occurs among immunodeficient patients [8]. *T. gondii* is a protozoan parasite with infection of humans occurring via consuming food or water contaminated with oocysts excreted in cat feces or bradyzoites-containing cysts in raw or poorly cooked meat [8]. Based on experimental work, different possible mechanisms have been postulated that the parasite may employ to cross the blood-brain barrier. One way is paracellularly or transcellularly through endothelial cells by involving EGFR as a possible molecular mechanism [9]. Alternatively, the parasite may use immune cells such as macrophages, neutrophils, dendritic cells, and monocytes [10–12].

The potential association between *T. gondii* infection and the development of brain tumors, particularly within the sellar region, has gained attention in previous literature [13–15]. Instances of sellar toxoplasmosis concurrent with pituitary adenomas are exceedingly rare, with only three documented cases reported this far [13, 15]. Notably, two of these cases involved immunocompetent patients with prolactinomas. It has been postulated that certain pituitary gland cells may proliferate, leading to increased prolactin production, supported by evidence indicating that exogenous prolactin can trigger antiparasitic activity in microglial cells [16]. In addition, B and T lymphocytes, as well as macrophages, possess prolactin receptors, whose activation can stimulate the secretion of cytokines such as tumor necrosis factor, interferon *γ*, and interleukin 12 [17]. Several studies have demonstrated the modulatory effects of prolactin on *T. gondii* proliferation and frequency [18, 19]. Further exploration into the interplay between *T. gondii* infection and prolactin signaling pathways holds promise for elucidating the pathogenesis in cases lacking traditional risk factors.

A case study by Berkmann et al. reported the presence of toxoplasmosis cysts within an inactive pituitary adenoma in an immunocompetent patient [15]. However, in our patient, who was also immunocompetent, histological examination did not reveal the presence of an adenoma. This raises the question of whether an adenoma was present but undetectable as it became necrotic. This case marks the fourth documented instance in the literature of *Toxoplasma* infection within the pituitary gland, all of which occurred in patients devoid of identifiable risk factors or systemic diseases, indicating a localized infection within the pituitary gland. This suggests a localized infection within the pituitary gland, distinct from systemic manifestations. Further investigation into the relationship between *T. gondii* infection and pituitary pathology may provide valuable insights into the pathogenesis of such cases.

A comprehensive literature review revealed only 2 cases of culture growth of *T. gondii* [2]. It has previously been shown that also intracranial toxoplasmosis without evidence of direct radiological involvement of the pituitary gland can present as panhypopituitarism [20]. Previously, especially for patients with HIV-related immune deficits, serologic tests have been shown to have low predictive value [21, 22], serving as a possible explanation for our patient's blood samples. Seronegative ocular *Toxoplasma* panuveitis has also been reported in the literature [23].

## 4. Conclusion

We report, for the first time, a case of isolated *T. gondii* abscess of the pituitary gland in an immunocompetent patient. Pituitary abscess should be considered also in radiologically suspectable adenoma. One important lesson for the present case is that pituitary *T. gondii* abscess can also form in seronegative, immunocompetent patients.

## Data Availability

The data used to support the findings of this study are included in the article.
